# Cellular ATP Levels Determine the Stability of a Nucleotide Kinase

**DOI:** 10.3389/fmolb.2021.790304

**Published:** 2021-12-13

**Authors:** Oliver Brylski, Puja Shrestha, Patricia Gnutt, David Gnutt, Jonathan Wolf Mueller, Simon Ebbinghaus

**Affiliations:** ^1^ Institute of Physical and Theoretical Chemistry, TU Braunschweig, Braunschweig, Germany; ^2^ Braunschweig Integrated Centre of Systems Biology (BRICS), Braunschweig, Germany; ^3^ Institute of Physical Chemistry II, Ruhr University, Bochum, Germany; ^4^ Institute of Metabolism and Systems Research (IMSR), University of Birmingham, Birmingham, United Kingdom; ^5^ Centre for Endocrinology, Diabetes and Metabolism (CEDAM), Birmingham Health Partners, Birmingham, United Kingdom

**Keywords:** PAPS synthase, ATP depletion, in-cell spectroscopy, protein folding stability, alanine scanning, ligand binding, sulfation pathways, cellular stress

## Abstract

The energy currency of the cell ATP, is used by kinases to drive key cellular processes. However, the connection of cellular ATP abundance and protein stability is still under investigation. Using Fast Relaxation Imaging paired with alanine scanning and ATP depletion experiments, we study the nucleotide kinase (APSK) domain of 3′-phosphoadenosine-5′-phosphosulfate (PAPS) synthase, a marginally stable protein. Here, we show that the in-cell stability of the APSK is determined by ligand binding and directly connected to cellular ATP levels. The observed protein stability change for different ligand-bound states or under ATP-depleted conditions ranges from ΔG_f_^0^ = -10.7 to +13.8 kJ/mol, which is remarkable since it exceeds changes measured previously, for example upon osmotic pressure, cellular stress or differentiation. The results have implications for protein stability during the catalytic cycle of APS kinase and suggest that the cellular ATP level functions as a global regulator of kinase activity.

## Introduction

In eukaryotic cells, there is abundant ATP at millimolar concentrations (Traut, 1994). Under conditions of stress, such as starvation (Maddocks et al., 2013; Petrovska et al., 2014) or DNA damage (Bonora et al., 2012), the ATP concentration is fluctuating inside cells. Recently, ATP was attributed an additional role as a ‘biological hydrotrope’; it controls the solubility and stability of proteins and protein complexes and governs liquid-liquid phase separation (Patel et al., 2017; Hayes et al., 2018; Gnutt et al., 2019b).

A class of proteins traditionally linked to ATP are P-loop kinases; they are ATP-dependent phosphate-transferring enzymes (Leipe et al., 2003). Nucleotide kinases are a kinase sub-family-they have ATP and some other nucleotide as their substrates. A special class of nucleotide kinases are those that phosphorylate the atypical nucleotide adenosine-5′-phosphosulfate (APS). Because APS is created in an upstream reaction that strongly relies on ATP as a substrate (Mueller and Shafqat, 2013), all substrates and all products of the reaction catalyzed by APS kinase (APSK) directly or indirectly depend on ATP availability (Brylski et al., 2019).

APSK enzymes sit at the center of sulfation pathways, as they are essential to produce active sulfate in the form of 3′-phosphoadenosine-5′-phosphosulfate (PAPS) (Gunal et al., 2019). With more than 50 PAPS-utilizing sulfotransferases encoded in the human genome, for example generating heparan sulfate (Gesteira et al., 2021), defects in the supply of the active sulfate PAPS should result in multi systems-defects. Clinical mutations in the human APS kinase-containing enzyme PAPS synthase 1 (PAPSS1) have not been described so far. However, disease-relevant point mutations in PAPSS2 have been linked specifically to bone and cartilage mal-formation (Iida et al., 2013) as well as dysregulation of steroid hormone action (Noordam et al., 2009; Oostdijk et al., 2015; Mueller et al., 2018). Noteworthy, a previous report classified PAPSS2 as a marginally stable protein, when being studied as recombinant protein (van den Boom et al., 2012). It remains unclear whether this marginal stability and possibly misfolding and subsequent aggregation of disease-related mutant proteins is a cause of the diseases mentioned above (Brylski et al., 2019).

Alternatively, the marginal stability of the PAPSS2 enzyme could actually serve a biological role in regulating enzymatic activity in sulfation pathway (Brylski et al., 2019). PAPS synthase isoforms are only marginally stable when studied as recombinant proteins, but they are stabilized by binding of their natural nucleotide ligands (van den Boom et al., 2012; Brylski et al., 2019). Most proteins should be stabilized by ligand binding (Luque et al., 2002), which may also serve as a protective measure from degradation (Staniec et al., 2015). However, a high affinity of two binding partners and high binding energies do not necessarily result in a stabilization of the full protein structure, as observed very recently for the protein complex of human proteins histone H1 and prothymosin-α (Borgia et al., 2018). This demonstrates the necessity to study individual protein-ligand interactions and their effect on protein stability on a case-by-case basis. For PAPS synthases, the main stabilizing ligands are PAPS, ADP as well as APS (Mueller and Shafqat, 2013); they all preferentially bind to the APSK (van den Boom et al., 2012). The protein-ligand interactions and stability of the respective ligand-bound states were traditionally studied using recombinant proteins and in aqueous buffers. Hence, it remains unclear if the stabilization of APSK by ligand binding (van den Boom et al., 2012) is preserved inside crowded cells and how protein stability of the APSK kinase domain is affected by fluctuating cellular ATP levels.

Here, we measure the in-cell stability of the APS kinase domain (APSK) of PAPSS2 under different conditions, using *Fast Relaxation Imaging* in combination with the engineered FRET-based fluorescent folding sensor APSK37. By alanine-scanning mutagenesis, we probe the stability changes of the APS kinase domain in different ensembles of substrate bound states. The observed stability changes correlate with the type of interaction deleted and its impact on the catalytic cycle. This implicates a changing protein stability for different states of the catalytic cycle. Further, we monitor the stability of this kinase domain at different intracellular ATP levels to determine the impact of ATP on the regulatory mechanism of sulfation pathways. Here, the overall protein stability of this kinase is directly coupled to cellular ATP levels, with marginal stability at low cellular ATP levels. In summary, we demonstrate that the APS kinase domain undergoes a protein-stability cycle that is coupled to the different ligand-bound states during its catalytic cycle and cellular ATP levels.

## Materials and Methods

### Plasmid Preparation

Full-length human PAPS synthase 2b (NM_001015880) in a EGFP-C1 vector was described previously (Schroder et al., 2012). The APS kinase domain of PAPSS2 was subcloned into a modified pDream2.1 vector with an N-terminal AcGFP1 and a C-terminal mCherry by PCR (In-Fusion, Clontech). The AcGFP1 is additionally tagged with an 6-His-Tag for protein purification. Point-mutations were introduced using site-directed mutagenesis (QuikChange Lightning, Agilent Technologies). Plasmid DNA was amplified in NEB5α (New England Biolabs), Stellar (Clontech) or XL10 Gold (Agilent Technologies) competent *E. coli* cells and purified using Zyppy Miniprep plasmid preparation kits (Zymo Research). DNA was quantified by UV/Vis spectroscopy (NanoDrop 2000; Thermo Fisher) and sequenced at an intramural facility (RUB Bochum).

### Protein Purification

NiCo21(DE3) competent *E. coli* (New England Biolabs) were transformed with plasmid DNA. A single colony was grown at 37°C and moderate shaking at 220 rpm to OD_600_ 0.6 in LB broth medium and then induced by addition of 100 µM IPTG. Protein expression was allowed overnight (∼16 h) at 18°C. Cells were harvested via centrifugation and lysed using xTractor buffer (Clontech). Protein suspension was transferred onto gravity flow His60 Ni Gravity Flow columns (Clontech) and purified according to the manufacturers protocol. Buffer was exchanged using Amicon Ultra (MWCO 30 kDa) to PBS (137 mM NaCl, 2.7 mM KCl, 10 mM Na_2_HPO_4_, 1.8 mM KH_2_PO_4_) at pH 7.4. Proteins were aliquoted and shock-frozen for long-term storage. Purity was evaluated via SDS-PAGE.

### Cell Culture and Plasmid Transfection

HeLa cells were grown in DMEM supplemented with 10% FBS, 100 U/ml penicillin and 0.1 mg/ml streptomycin as an adherent culture (Sarstedt) in a humidified atmosphere (37°C, 5% CO_2_). Cells were passaged in a 1:4 or 1:6 ratio after 2 to 3 days upon reaching 80–90% confluence using trypsin digestion. For experimental preparation, cells were seeded on six-well plates (Sarstedt) and transfected at 80–90% confluence using Lipofectamine 3,000 (Thermo Fisher) according to the manufacturers protocol. Briefly, 125 µl Opti-MEM (Thermo Fisher) was supplemented with 2 µg of according plasmid DNA and 4 µl P3000 reagent. Mixture was transferred and mixed with 125 µl Opti-MEM supplemented with 4 µl Lipofectamine3000 reagent after 5 min of incubation. Transfection mixture was added to the cellular growth medium and cell culture incubated for 6 hours. After incubation, cells were passaged using trypsin digestion and seeded on 35 mm glass bottom dishes (Fluorodish, World Precision Instruments) and grown for 2 days at regular cell culture conditions.

### Sample Preparation

In-cell measurements using Fast Relaxation Imaging were performed with transfected cells grown on 35 mm glass bottom dishes (Fluorodish, World Precision Instruments). Growth medium was aseptically removed and cells washed with DPBS (Sigma-Aldrich). Glass bottom dishes with cells were placed on a glass cover slip (Menzel #1.0) with a 120 µm thick imaging spacer (Sigma-Aldrich) and covered with 30 µl Leibovitz’s L15 medium supplemented with 30% FBS. For measurements at ATP depleted conditions, Leibovitz’s L15 medium was supplemented with 1 mM KCN (Sigma-Aldrich) and 10 mM 2-deoxyglucose (Sigma-Aldrich) and cells incubated for the respective time prior to the FReI measurement.

Measurements in buffered solutions were performed at 10 µM protein concentration in PBS supplemented with 10 µM MgCl_2_. Nucleotides were dissolved in PBS and added at desired concentration. 20 µl of sample were sealed between a glass cover slip with a 120 µm thick imaging spacer and a 35 mm glass bottom dish.

HeLa cells transfected with the ATeam1.03-nD/nA protein sensor were not sealed as the ATP depleting agents had to be supplemented during the measurement.

### Fast Relaxation Imaging Measurements

Fast Relaxation Imaging combines millisecond temperature jumps with wide field fluorescence microscopy (Ebbinghaus et al., 2010). The microscope setup as well as the fluorophore pair used in this study have been described and characterized earlier (Dhar et al., 2011; Gao et al., 2016; Buning et al., 2017). Briefly, the specimen is rapidly heated by an IR diode laser (m2k-Laser, 2,200 nm) in the millisecond range while recording change of fluorescent signal using CCD cameras. The temperature jumps were calibrated using the temperature sensitive dye Rhodamine B (Vopel et al., 2015; Gao et al., 2016; Buning et al., 2017). The heating profile used throughout this study consisted of individual 2.2°C temperature jumps at intervals of 50 s (Supplementary Figure 1), covering a temperature range from 23.0°C to 58.2°C in 16 steps. Images were recorded at a frame rate of 1 fps and image acquisition times were between 50–200 ms with LED exposure only during acquisition times. At least three technical replicates (N ≥ 3), including separate cell culturing and measuring, were performed for in-cell measurements. For *in vitro* measurements at least three technical replicates were performed (N ≥ 3).

Images were processed and evaluated using ImageJ (National Institute of Health, United States). Retrieved intensity data were further evaluated using self-written MatLab (Mathworks) codes and GraphPad Prism 6 (GraphPad). The cytoplasmic region of the cell was defined by excluding the visible nuclear area and fluorescence intensity averaged throughout this region of interest for each channel individually. *In vitro* samples were averaged throughout a defined region covering about 90% of the image.

After subtraction of the background signal for the individual donor (D) and acceptor (A) channels, the ratio D/A was calculated for an initial evaluation of data and *D-αA* for kinetic analysis (Dhar et al., 2011). Single temperature jumps and their unfolding kinetics were fitted to single exponentials, reflecting a two-state folding behavior, and the kinetic amplitudes (*D-αA(T)*) plotted against temperature. These amplitudes were then fitted to the thermodynamic model introduced as *Better thermodynamics from kinetics* (Girdhar et al., 2011):

$$D-\alpha A\left(T\right)=\frac{-\delta g_{1}T\Delta \cdot T_{M}}{R{\left(T-\Delta T/2\right)}^{2}}\cdot \left(A_{0}+m_{A}\left(T-T_{M}\right)\right)\cdot \frac{\text{exp}\left(-\delta g_{1}\left(T-\left(\Delta T/2\right)-T_{M}\right)\cdot {\left(R\left(T-\Delta T/2\right)\right)}^{-1}\right)}{{\left(1+\text{exp}\left(-\delta g_{1}\left(T-\left(\Delta T/2\right)-T_{M}\right)\cdot {\left(R\left(T-\Delta T/2\right)\right)}^{-1}\right)\right)}^{2}}$$

The determined fitting parameters are *δg*_*1*_ and *T*_*M*_. *δg*_*1*_ is the pre-factor of the linear Taylor approximation of the two-state population and *T*_*M*_ the melting point of the protein unfolding transition analyzed. *ΔT* is the amplitude of the temperature (set to 2.2°C) and *A*_*0*_ and *m*_*A*_ the fitting parameters of the underlying baseline (with *m*_*A*_ set to 0). The melting point *T*_*M*_ was compared for individual point-mutants measured. Fits were performed using GraphPad Prism 6.

The pre-factor of the linear Taylor approximation *δg*_*1*_ as well as *T*_*M*_ are used to calculate the standard free energy of folding (Dhar et al., 2011; Girdhar et al., 2011):

$$\Delta G_{f}^{0}=-\delta g_{1}\left(T_{M}-T\right)$$

with *T* being the reference temperature of 37°C or 310.15 K respectively.

The results of the different solvated alanine mutants need to be compared to the wildtype APSK37 sensor as an internal standard and depict a lower limit of the actual thermodynamic contribution.

### ATP Depletion Experiments

The ATeam1.03-nD/nA protein sensor used for depletion experiments is based on the ε-subunit of the bacterial F_0_F_1_-ATP synthase (Kotera et al., 2010). Recordings of ATP depletion were performed using an Olympus FV3000 CLSM confocal microscope. Transfected HeLa cells were supplemented with 1 mM KCN and 10 mM 2-deoxyglucose during the measurement and the solution carefully mixed using a pipette. The sensor was excited using a 445 nm laser and the signal recorded via an UPLXAPO ×20 objective (NA 0.8, Olympus). Donor and acceptor signals were detected on separate detectors for 50 min in 5 s intervals. Two technical replicates (N = 2), including new passaging of cells and transfection, were performed to determine relative ATP concentration changes during treatment.

## Results

### Determining the in-Cell Stability of APS Kinases *in vitro* and in the Cell

**FIGURE 1:**
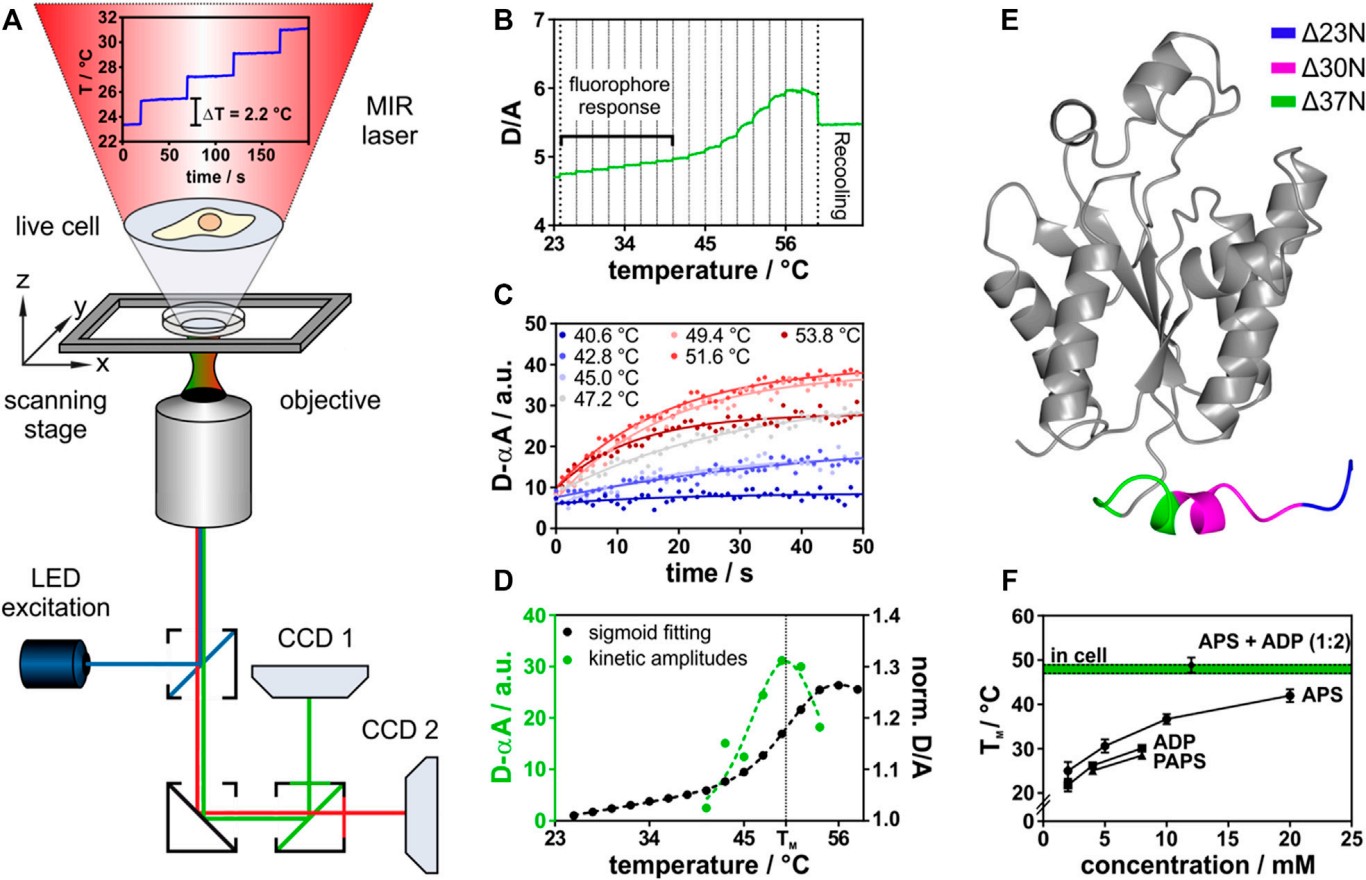
In-cell thermal unfolding of APSK37 using Fast Relaxation Imaging. **(A)** Representation of the Fast Relaxation Imaging setup. **(B)** Thermal unfolding curve of APSK37. Bold dotted lines represent start and end-point of T-jumps applied. Faint dotted lines represent start-point of each single T-jump. **(C)** Kinetics of APSK37 unfolding at the respective temperatures. **(D)** Comparison of sigmoid fitting of the thermal unfolding data to the *Better Thermodynamics from Kinetics* method. **(E)** Crystal structure of human PAPS synthase 2 APS kinase domain (PDB: 2AX4). Truncations of the N-terminal tail region are highlighted in color. **(F)** *In vitro* ligand titration of APSK37 (10 µM). Total concentration of both nucleotides APS and ADP is at the indicated 1:2 ratio (8 mM ADP, 4 mM APS). Data points represent mean ± s.d and include at least three replicates (N ≥ 3). For comparison, the APSK37 in-cell melting temperature is shown in green. Dotted lines indicate ±s.d. of mean.

In order to study protein-ligand interactions and their effects on protein stability in the complex environment of the cell, we use *Fast Relaxation Imaging*. It is a unique tool to study protein folding kinetics and thermal stability in single living cells with high spatial and temporal resolution (Ebbinghaus et al., 2010; Dhar et al., 2011; Wirth et al., 2013). *Fast Relaxation Imaging* combines wide-field fluorescence microscopy with consecutive temperature jumps utilizing a mid-infrared laser and Förster Resonance Energy Transfer (FRET) (Figure 1A). Analysis of kinetic amplitudes of protein unfolding at the respective temperatures allows to determine the thermal stability of the protein in the cell (Dhar et al., 2011; Girdhar et al., 2011).

In this study, a temperature profile with 16 consecutive temperature jumps was used. This resulted in individual temperature jumps of 2.2°C covering a temperature range from 23.0°C to 58.2°C (Supplementary Figure 1). The consecutive temperature jumps allowed to measure the thermal melting curve of a FRET-labeled APS kinase (Figure 1B) within 16 min. The respective unfolding kinetics at each temperature jump were fitted (Figure 1C) using the *Better Thermodynamics from Kinetics* method (Girdhar et al., 2011) (see Material and Methods for details). The amplitudes of the unfolding kinetics were analyzed, showing a maximum at the melting temperature (T_M_) due to the highest population shift between folded and unfolded states. In common fitting of thermal melting curves folded and unfolded baselines need to be resolved, which does not need to be accounted for in this model (Figure 1D), making the fitting procedure more robust (Girdhar et al., 2011). The standard free energy of folding at 37°C (ΔG_f_^0^) was then determined by a linear Taylor approximation as described earlier (Dhar et al., 2011; Girdhar et al., 2011).

This method was applied both in aqueous buffered solution *in vitro* and in the cell under the same experimental conditions. For FRET detection, we constructed a fusion protein of the APS kinase domain of human PAPS synthase 2 (Figure 1E). The APS kinase domain catalyzes the rate-limiting step in PAPS synthesis (Grum et al., 2010) and harbors four of the six known disease-mutations (Kurima et al., 1998; Noordam et al., 2009; Iida et al., 2013). To construct the fusion protein, we truncated the two-domain bifunctional PAPSS2 protein between two isoleucine residues (I220 and I221) within the flexible linker region connecting the kinase and sulfurylase domain (Harjes et al., 2005). In bacteria, fungi and plants, APS kinase and ATP sulfurylase are not observed as fusion proteins (Patron et al., 2008) and crystal structures (Sekulic et al., 2007a) as well as enzymology studies (Harjes et al., 2005; Sekulic et al., 2007a) indicate that the isolated APS kinase remains fully functional, rationalizing the choice of truncation for the experiment. Next, we step-wise truncated the flexible and disordered N-terminal region. An APS kinase variant, shortened by 37 amino acids (Δ37N), resembles a truncation variant of the PAPSS1 homolog that retained catalytic activity (Sekulic et al., 2007a). Only this variant allowed us to study the conformational dynamics of the monomer by intramolecular FRET using AcGFP1 (N-terminus) and mCherry (C-terminus) as fusion proteins (Supplementary Figure 2). We refer to this newly engineered folding reporter as APSK37 from now on.

APSK37 showed an intrinsic donor (D) to acceptor (A) fluorescence intensity increase upon IR-laser heating (Figure 1B, fluorophore response), resulting from the different temperature dependency of quantum efficiencies of the FRET pair AcGFP1 and mCherry (Dhar et al., 2011). Thermal unfolding was then observed at T_M_ = 48.0 ± 1.7°C giving rise to an unfolding curve with a single transition. The unfolding process is reversible for a fraction of the protein ensemble as indicated by recovery towards the initial D/A ratio upon temperature relapse (Feng et al., 2019) (Supplementary Figure 3). Unfolding kinetics at the respective temperatures show single exponential behavior, suggesting two-state unfolding (Figure 1D).

For *in vitro* experiments, we purified the recombinant APSK37 fusion protein from *E. coli* to investigate its substrate binding capacity (see Material and Methods). Reversible unfolding and refolding of the protein *in vitro* requires the addition of nucleotides (Supplementary Figure 4), illustrating their role in determining folding stability. Titrations of the recombinant APSK37 protein with different nucleotides showed a pronounced stabilization by APS followed by a less pronounced stabilization by ADP and PAPS (Figure 1F) which is in good agreement with previous *in vitro* studies of full-length PAPS synthase 2 (van den Boom et al., 2012). Combination of the nucleotides ADP and APS at a ratio of 2:1, as found in nucleotide-soaked crystals of the PAPSS1 APS kinase domain (PDB: 2PEY), stabilized the protein to a much larger extent than addition of only one nucleotide alone. In fact, joint binding of ADP and APS nucleotides to APSK37 resulted in similar T_M_ values to the ones determined inside live cells (Figure 1F) (48.0 ± 1.7°C compared to 48.9 ± 1.7°C). Experiments in buffered solutions neglect excluded-volume (Zimmerman and Trach, 1991) and non-specific binding (McConkey, 1982) present inside crowded cells, however these results show that both nucleotide binding sites of this kinase need to be occupied to compensate for being naturally fragile (van den Boom et al., 2012).

### APSK37 in-Cell Thermal Stability is Determined by Ligand Binding

**FIGURE 2:**
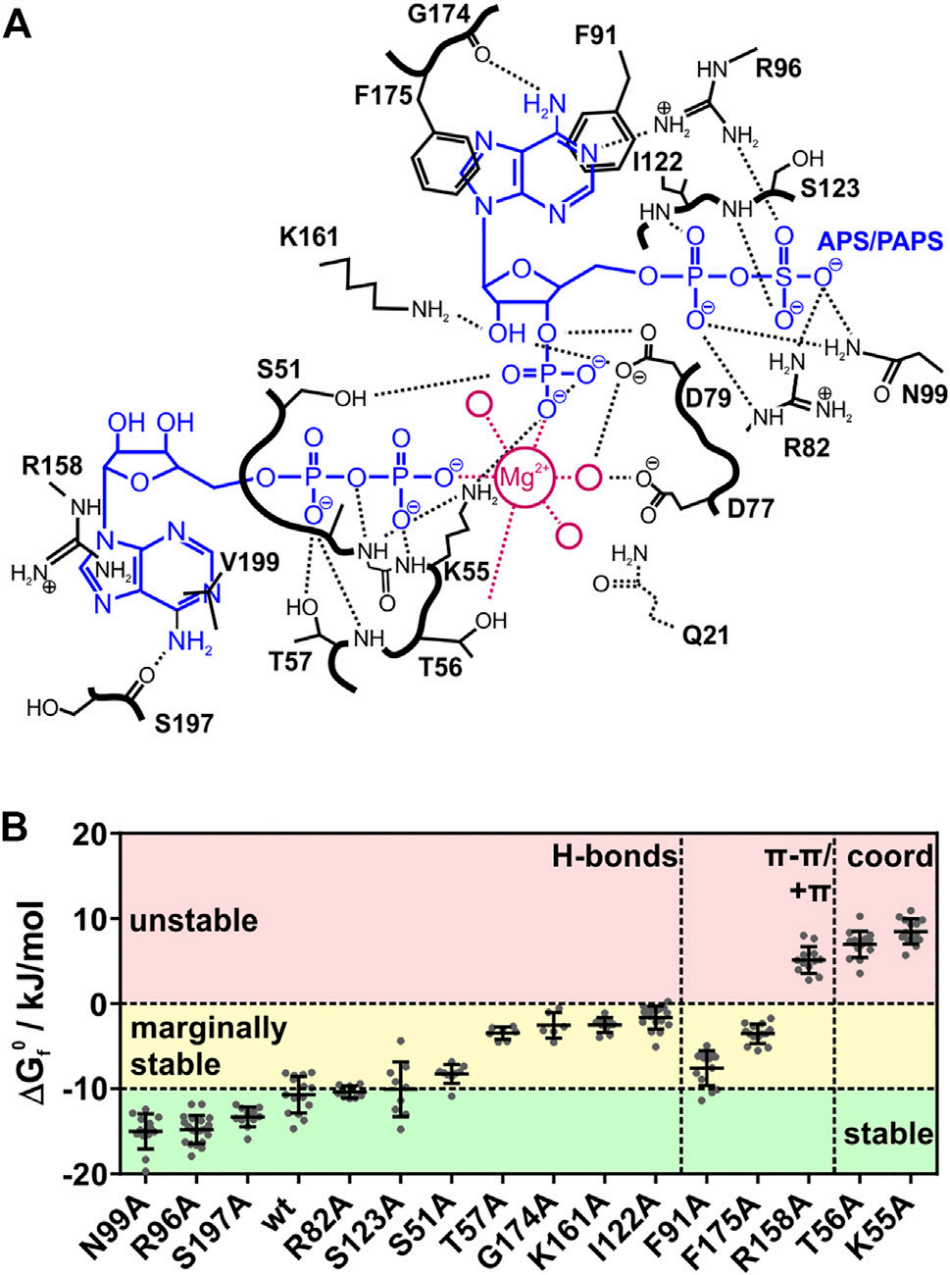
Interactions and stability of APSK37 point-mutants. **(A)** Contact map of the ADP/ATP and APS/PAPS binding site of the PAPSS1 APS kinase domain adapted from *Sekulic et al.* (PDB: 2PEZ). Magenta colored circles represent water molecules coordinating the magnesium cation. Bold black lines represent the peptide backbone. Q21 was deleted in APSK37 **(B)** Standard free energy of folding at 37°C (ΔG_f_^0^) for in-cell measured APSK37 binding site mutants. Mutations with a ΔG_f_^0^ lower −10 kJ/mol are referred to as stable. Mutations showing ΔG_f_^0^ of −10 kJ/mol to 0 kJ/mol are referred to as marginally stable. Mutations showing ΔG_f_^0^ larger 0 kJ/mol are referred to as unstable. Data points depict single cells measured. Bars refer to mean ± s.d.

Next, we used APSK37 to elucidate the influence of substrate, product and co-factor binding on protein stability inside cells, by systematically alanine-scanning its ligand binding sites. We know that ADP, APS and PAPS stabilize the PAPS synthases proteins (van den Boom et al., 2012). Analogous in-cell nucleotide titrations are not feasible as nucleotide concentrations cannot be controlled inside cells in a quantitative manner. To introduce changes in binding affinity of the protein to its nucleotide ligands and the metal co-factor, we probed the protein environment of the ATP/ADP and the APS/PAPS nucleotide binding sites as well as the P-loop coordinating the Mg^2+^ cation by alanine scanning mutagenesis (Figure 2A, see Table 1). Binding sites of the APS kinase domain of human PAPS synthases have been characterized structurally before (Harjes et al., 2005; Rabeh et al., 2005; Sekulic et al., 2007a). This allowed us to selectively delete different types of chemical contacts, such as hydrogen bonds, π-π- or cation-π-stacking interactions or cation coordination (see Table 1). Deletion of the stronger interactions, such as cation coordination or stacking interactions, is expected to reduce affinity more than deleting a single H-bond. This approach enabled us to evaluate the in-cell stability change of APSK37 in an indirect way that is comparable to classic titration experiments with recombinant proteins and different ratios of bound and unbound substrate ensembles.

**TABLE 1:** Summary of APSK37 in-cell binding-site analysis. Mutations are split into stable, marginally stable and unstable variants and sorted according to their ΔG_f_^0^. ΔG_f_^0^ corresponds to the standard free energy of folding at 37°C. Asterisks depict mutations originally interacting with the nucleotide *via* their peptide backbone. Values are depicted as mean ± s.d.

Mutation	Interaction	Nucleotide	Partner	T_M_/°C	ΔG_f_^0^/kJ/mol	n
**Stable**						
N99A	H-bond	APS/PAPS	α-phosphate/β-sulfate	47.8 ± 0.6	-15.1 ± 2.1	13
R96A	H-bond	APS/PAPS	β-sulfate/adenine base	50.5 ± 0.6	-14.8 ± 1.6	20
*S197A	H-bond	ATP/ADP	adenine base	49.8 ± 0.6	-13.3 ± 1.2	11
Wt	---	---	---	48.0 ± 1.7	-10.7 ± 2.2	15
R82A	H-bond	APS/PAPS	α-phosphate/β-sulfate	47.7 ± 1.9	-10.4 ± 0.7	8
*S123A	H-bond	APS/PAPS	α-phosphate/β-sulfate	48.6 ± 3.3	-10.1 ± 3.2	9
**Marginally stable**						
S51A	H-bond	APS/PAPS	3′-phosphate	46.4 ± 1.3	-8.3 ± 1.1	9
F91A	π-π	APS/PAPS	adenine base	47.1 ± 2.7	-7.6 ± 2.0	13
F175A	π-π	APS/PAPS	adenine base	40.9 ± 2.3	-3.5 ± 1.1	14
T57A	H-bond	ATP/ADP	α-phosphate	40.9 ± 1.1	-3.4 ± 0.7	7
*G174A	H-bond	APS/PAPS	adenine base	38.4 ± 0.7	-2.5 ± 1.5	6
K161A	H-bond	APS/PAPS	4′-hydroxyde	39.5 ± 1.4	-2.5 ± 0.9	9
*I122A	H-bond	APS/PAPS	α-phosphate	38.9 ± 1.9	-1.6 ± 1.4	17
**Unstable**						
R158A	+π	ATP/ADP	adenine base	33.2 ± 1.3	5.1 ± 1.6	12
T56A	+coord	---	Mg^2+^ cation	29.2 ± 0.9	7.0 ± 1.5	15
K55A	+coord via H-bond	ATP/ADP	β-phosphate/3′-phosphate	28.1 ± 0.8	8.5 ± 1.5	14
		APS/PAPS				

We determined the in-cell melting temperature T_M_ as well as the standard free energy of folding at 37°C (ΔG_f_^0^) of the respective alanine mutations. Mutants showing a ΔG_f_^0^ energy of less than −10 kJ/mol are referred to as stable mutants. Mutants with ΔG_f_^0^ energies between −10 kJ/mol and 0 kJ/mol are referred to as marginally stable mutants, in accordance with a recent definition (Pastore et al., 2019), and mutants with a ΔG_f_^0^ energy of more than 0 kJ/mol are referred to as unstable (Figure 2B, see Table 1).

Alanine-scanning of hydrogen bonding, which we expected to have a small impact on affinity changes upon deletion, showed largely varying protein stabilities. The stable mutants R96A, N99A and S197A show ΔG_f_^0^ values in a range of −15 to −13 kJ/mol, being more stable than the wild type. Mutants R82A and S123A do not change in their ΔG_f_^0^ values from the wild type. Here, the hydrogen bonds are formed via the peptide backbone or the side-chain with either the β-sulfate of APS/PAPS or the adenine bases of the nucleotides. The deletion of their interactions does seemingly not alter the affinity of the protein to its ligands and co-factor.

However, alanine variants with deleted hydrogen bonds are also found in the marginally stable energy range. Mutation S51A showed a minor destabilization with a difference of 2.4 kJ/mol to the APSK37 wild type. Mutations T57A, I122A, K161A and G174A represent a more destabilized, but still marginally stable group at physiological conditions indicated by the negative ΔG_f_^0^, ranging from −3.4 to −1.6 kJ/mol. S51 interacts with the 3′-phosphate of PAPS, T57 interacts with the α-phosphate of ATP/ADP and I122 with the one of APS/PAPS via its peptide backbone, K161 with the ribose 4′-hydroxide of APS/PAPS and G174 *via* its peptide backbone with the adenine base of APS/PAPS. Surprisingly, the peptide backbone interactions with the α-phosphate and APS/PAPS adenine base via I122 and G174 result in a pronounced destabilization, which we did not expect to occur from exchanges in the side-chains. The effects must therefore be connected to the gain (I122A) or loss (G174A) of flexibility and the consequences for the structural integrity of the APS/PAPS binding-site. Here, the interactions of marginally stable H-bonds residing close to the center of catalysis, the transfer of the γ-phosphate of ATP onto the 3′ hydroxide of APS, are highly stabilizing. Hence, the effect of hydrogen bonds appears to depend on the localization within the protein.

Deleted stacking interactions, occurring either as π-π stacking with the adenine base of APS/PAPS (F91, F175) or cation-π stacking with the ATP/ADP adenine base (R158), range from marginally stable to unstable. Deletion of π-π-interactions differently impacts on the destabilization comparing the ΔG_f_^0^ values of −7.6 kJ/mol (F91A) and −3.5 kJ/mol (F175A). However, the loss of π-π interactions is overall destabilizing, making the protein marginally stable. Deletion of cation-π stacking in the ATP/ADP binding site resulted in a severe destabilization, shifting the APSK37 protein from a stable protein to an unstable one by deletion of a single protein-ligand interaction.

The cation coordination of the p-loop mutants T56 and K55 is of similar importance as the stacking interaction of R158, indicated by ΔG_f_^0^ values ranging from 7.0 to 8.5 kJ/mol. According to the pronounced destabilization compared to wild-type APSK37, the cation coordination within the p-loop in a direct (T56) or indirect manner via orientation of the nucleotides phosphates (K55) (Figure 2A) is crucial for protein stability. These data suggest that cation coordination within APSK37 is essential for the protein to fold.

Next, we analyzed initial and final D/A values in our experiments that are representative of the initial and final conformational states of the different mutants (Supplementary Figure 5). Folded mutants (stable and marginally stable) show largely identical initial D/A values, indicative of a similar folded fraction of protein at the beginning of the experiment (Supplementary Figure 5). The exceptions within the folded mutants are N99A and G174A. N99A shows low initial D/A values prior to the temperature jumps and similar final D/A values after temperature relaxation compared to other mutants. G174A shows low initial as well as final D/A values. This exception may be attributed to protein oligomerization prior to the experiment. Still, unfolding transitions could clearly be observed for this mutant.

In contrast to the folded mutants, we found that the unstable mutants K55A, T56A and R158A showed decreased initial (3.5 compared to 4.5 for wild-type) and final D/A values (3.5 compared to 5.2 for wild-type) (Supplementary Figure 5). Further, the determined in-cell ΔG_f_^0^ is smaller than 0 kJ/mol (5.1–8.7 kJ/mol), reflecting proteins which are unfolded at physiological conditions. The final D/A decreased below the initial ratio and the negative D/A response at elevated temperatures for these three mutants due to intermolecular FRET (Ebbinghaus et al., 2010; Buning et al., 2017) (Supplementary Figure 5, 6; see negative kinetic amplitudes at T larger 36°C) indicates aggregation of the respective mutants prior and during the experiment at high temperatures. Again, unfolding transitions were still observable clearly for these mutants.

We could correlate the observed changes in standard folding free energy ΔG_f_^0^ due to the type and localization of the deleted protein-ligand interaction with the ligand titrations of the recombinant APSK37 protein (Figure 1F). Thus, we could estimate qualitatively how the population of the enzyme devoid of substrates and possibly co-factor, single-bound and fully occupied APSK37 shifted within the cell. For the stable mutants (see Table 1), the population at the beginning of the in-cell experiment should be predominantly on the side of occupied APSK37, as stability is not affected for the stable mutants. The deletion of their interactions seemingly does not alter the affinity of the protein to its ligands and co-factor. For the marginally stable mutants (see Table 1), the ensemble of APSK37 binding states should shift towards less stable states due to a major loss in affinity. Except for T57A, all mutations are located within the APS/PAPS binding site. Therefore, the single-bound state with only the ATP/ADP binding site occupied and the Mg^2+^ cation coordinated should most likely reflect the marginally stable mutants. For the unstable mutants (see Table 1), the population of APSK37 binding-states should shift to predominantly being the state devoid of substrates. Considering the type and localization of the interactions, the loss of coordination sites for the Mg^2+^ cation does largely affect ligand binding, resulting in low affinity of all binding sites, to their respective binding partner including the cation itself.

Here we have shown by alanine-scanning that nucleotide ligands and the metal co-factor binding determine APSK37 stability in living cells at physiological substrate and co-factor concentrations. Most deleted H-bonds do not shift populations of different ligand-binding states notably towards unstable states free of bound ligands. Deletion of stronger interactions, such as π-π or cation-π interactions however shift the APSK37 complex towards the substrate-free state, as noted by the pronounced destabilization. The largest effects were observed for deleted interactions necessary for cation-coordination, being the most crucial contacts to stabilize APSK37 upon ligand binding. Strikingly, the deletion of a single interaction is connected to the expected loss in affinity and results in pronounced protein destabilization, due to less protein population in ligand bound states.

### ATP Depletion Strongly Destabilizes the APSK37 Kinase Domain

We have shown how changing the binding affinity of all ligands mutating their binding sites impacts on APSK37 stability. The catalytic cycle of PAPS synthases shows that the key determinant for the availability of the stabilizing ligands, APS, PAPS and ADP, is ATP availability. The cellular ATP concentration, however, is prone to changes during stresses such as DNA damage (Dmitrieva et al., 2011) or cellular starvation (Maddocks et al., 2013; Petrovska et al., 2014). Depending on the nutrition or even the organelle investigated the ATP concentration may range from 8–10 mM to a low micromolar level (Imamura et al., 2009). Especially in active neurons, the ATP:ADP ratio can change on a minute timescale upon excitation from 40 to 1 (Tantama et al., 2013). For comparison, the half-live of PAPS synthases in different cell types can be up to 100 h (Mathieson et al., 2018) and therefore the APS kinase domain is necessarily affected by changing ATP concentrations due to cellular events. Therefore, we investigated how cellular ATP levels influence APSK37 stability.

**FIGURE 3:**
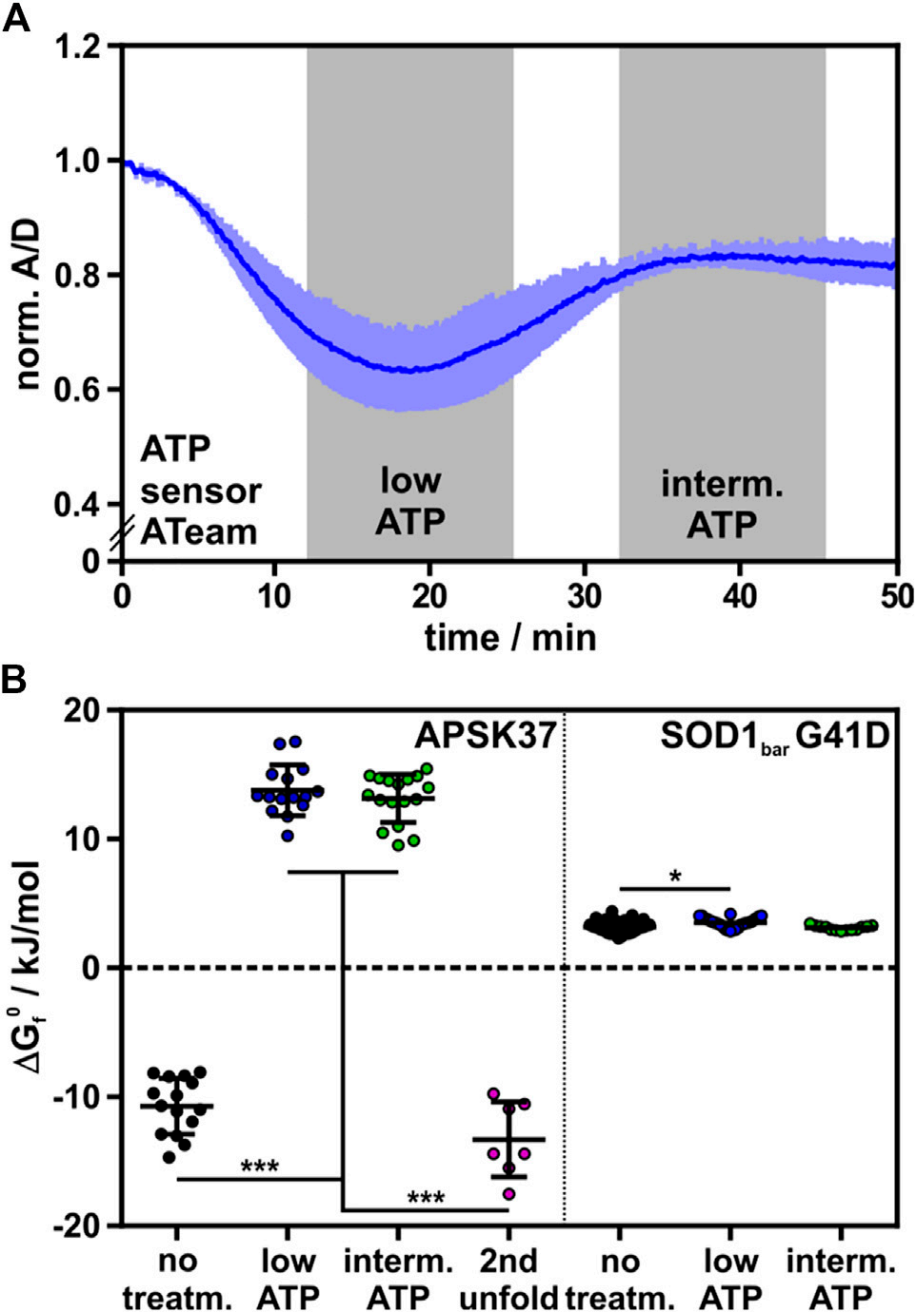
Effect of ATP depletion on APSK37 in-cell stability. **(A)** Normalized A/D signal of ATP sensor Ateam 1.03-nD/nA during ATP depletion of HeLa cells with 1 mM KCN and 10 mM 2-deoxyglucose. Time frames of low ATP and intermediate ATP concentration are highlighted in grey. Measurements including folding-reporters were performed within these time frames. Bars reflect s.d. (n = 3) **(B)** Standard free energy of folding at 37°C (ΔG_f_^0^) of APSK37 and SOD1_bar_ G41D (non-binding control) during ATP-depleting conditions. Significant differences between data sets were tested using a non-parametric Kruskal-Wallis test followed by a *post-hoc* Dunn’s test for multiple comparisons (***p < 0.001, *p < 0.05). Data points refer to single cells measured (exact numbers can be found source file of Figure 3B). Bars refer to mean ± s.d.

We depleted HeLa cells of ATP, using potassium cyanide (KCN) and 2-deoxyglucose (2DG), to poison the oxidative phosphorylation machinery and to clear the ATP pool available for APSK37 ligand binding and nucleotide ligand synthesis (Imamura et al., 2009). The change in cellular ATP during the treatment was detected using the FRET-based ATP protein-sensor ATeam (Kotera et al., 2010). The relative ATP concentration is reported as the acceptor to donor ratio (A/D) with decreasing ratios showing reduced FRET-efficiency and release of ATP from the sensor due to decreasing cellular concentrations. Incubation with KCN and 2DG led to a rapid decrease of ATP levels inside cells followed by a regeneration phase reaching an intermediate ATP level (Figure 3A) as described by *Imamura et al.* (Imamura et al., 2009). In-cell protein stability of APSK37 was measured before and after depletion of ATP, representing high, low and intermediate ATP levels (Figure 3A).

Stability of wild-type APSK37 at low and intermediate cellular ATP levels did not differ significantly. Compared to untreated cells, APSK37 is destabilized by 24.5 ± 2.9 kJ/mol at low cellular ATP levels and 23.9 ± 2.9 kJ/mol for intermediate ones. The observed range of destabilization exceeded the effect of the most severe alanine mutants, which showed a destabilization up to 19.2 ± 2.7 kJ/mol for APSK37 variant K55A (Figures 2B, 3B). The pronounced destabilization at lowered ATP levels may be attributed to a depletion of APS and PAPS as both nucleotides are generated from ATP. At low and intermediate ATP, the accessible pool of nucleotide binding partners appears to be mostly depleted, resulting in highly destabilized APSK37 within cells.

**FIGURE 4:**
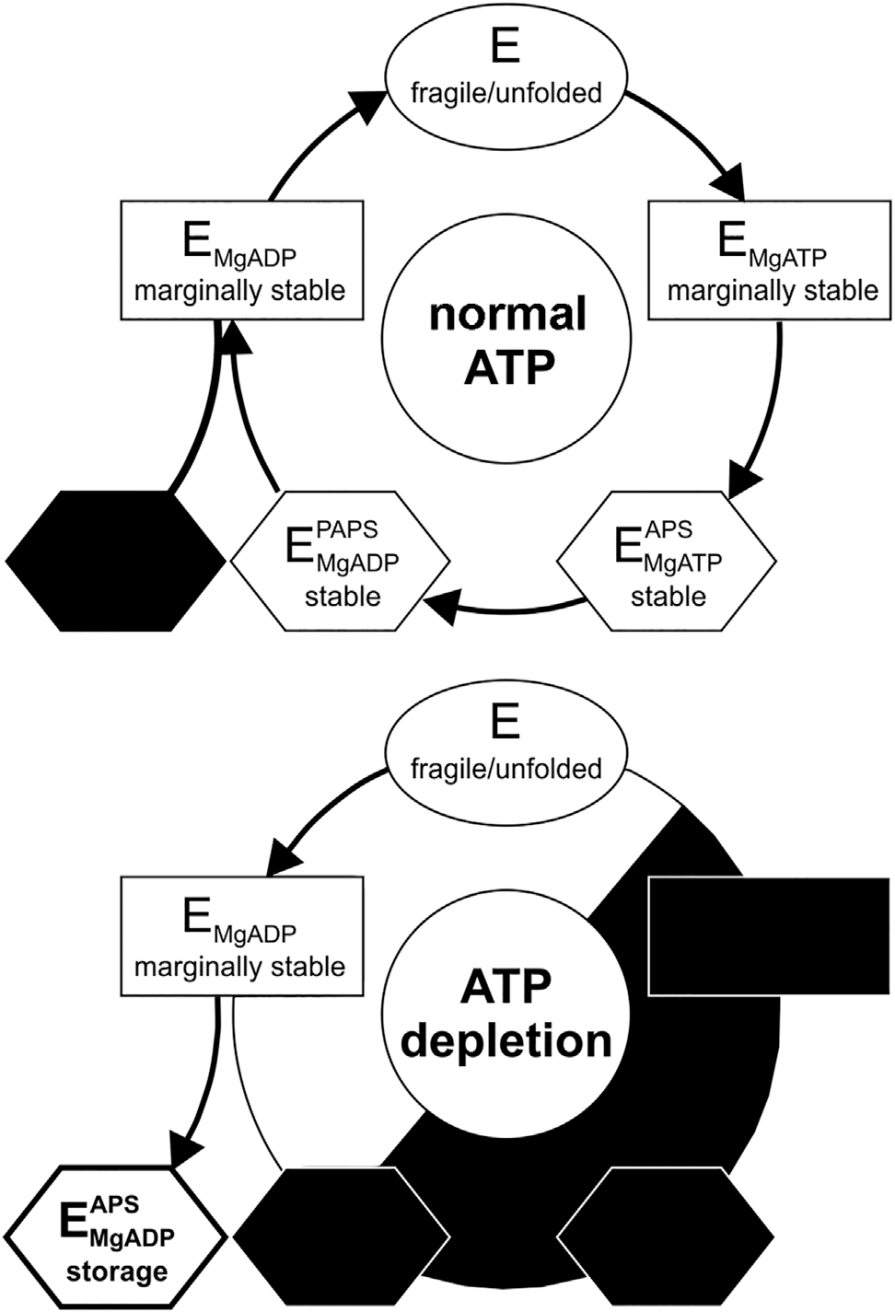
Catalytic and temporal stability cycle of APS kinase from PAPS synthase. (Top) Catalytic cycle of the APS kinase domain of PAPS synthases at physiological ATP concentrations. The storage form (ADP-APS complex) is most likely not significantly utilized under these conditions (blacked out) (Bottom) Distribution of the states during ATP depletion. Decreasing ATP concentration via starvation or DNA damage results in population of the kinase complexes within the cycle with the least phosphorylation equivalents of the substrates. The storage form of the PAPS intermediate APS and ADP may serve as a stable state for a fraction of the APS kinase protein ensemble in times of ATP depletion.

In addition to the low-temperature unfolding event described above, we observed a second high-temperature unfolding event within ATP-depleted cells, which shows similar ΔG_f_^0^ energies to the wild-type at regular ATP levels (Figure 3B); suggestive of two distinct protein populations in the observed protein ensemble. Unfolding at low temperatures resembles the unfolding of substrate-free APSK37 protein, best approximated by the K55A mutant. As the protein stability of the wild-type APSK37, which predominantly occurs in fully occupied binding states, is comparable to the high-temperature unfolding event, this state may be attributed to a fully occupied binding state as well. Lately, the inhibitory ADP-APS complex of APSK was proposed to may serve as a storage form in times of ATP depletion (Brylski et al., 2019). This suggests that the high-temperature event of unfolding, visible in ATP-depleted cells, may be connected to the highly stable ADP-APS-complex (Figure 4), which may protect the APSK from misfolding and aggregation.

We finally compared our measurements to the stability of an in-cell folding reporter that does not feature kinase activity or co-factor binding sites, the G41D mutation of the SOD1_bar_ protein (Gnutt et al., 2019b), as a control. We measured the stability of this folding reporter under the same experimental conditions and indeed found that this protein’s stability is insensitive to intracellular ATP concentration. We found only a minor destabilization at low ATP levels and no significant destabilization at intermediate ones (Figure 3B). This shows that not the treatment with KCN and 2DG itself, but the resulting depletion of ATP and consequently APS and PAPS is responsible for the severe destabilization of APSK37 in these measurements.

Taken together, ATP depletion caused a pronounced destabilization of the APSK37 wild-type kinase. Complementing the alanine-scanning that focused on site-directed reduction of affinity to the ligands, these data show that the stability of APSK37 is directly connected to the cellular ATP concentration and consequently the available nucleotide ligand pool.

## Discussion

Cells maintain abundant pools of ATP at millimolar concentrations (Traut, 1994). The necessity of such high ATP levels and their costly maintenance are not fully understood. For an enzyme like PAPS synthase where all stabilizing substrates need to be generated from ATP, stability of the enzyme is necessarily connected to the availability of ATP inside the cell. The nucleotide kinase domain of human PAPSS2, a naturally fragile protein, was the starting point for developing the FRET-based folding reporter APSK37. Using alanine-mutagenesis and ATP depletion experiments of APSK37 in living cells, we investigated the effect of ligand binding on APSK37 stability and how it is connected to cellular ATP levels.

By alanine-mutagenesis of amino acids interacting with the nucleotide ligands and the metal co-factor, the number of intermolecular interactions and ultimately affinity of APSK37 to either APS/PAPS or ATP/ADP is reduced. This allows to study the effect of ligand binding to APSK37 stability inside cells and to shift the ratios of the possible protein-ligand complexes formed towards single-bound or substrate-free form. Each alanine mutation was classified according to its ΔG_f_^0^ value (Figure 2B) into stable mutants, marginally stable (Pastore et al., 2019) and unstable proteins. The loss of hydrogen bonds to the nucleotide substrates results in stable and marginally stable protein. Noteworthy, half of the hydrogen bonds deleted (R82A, R96A, N99A, S123A and S197A) do not alter protein stability and most likely do not impair affinity, compared to an expected loss of binding energy of 4 kJ/mol (Davis and Teague, 1999). Alanine mutation of the other half resides close to the catalytic center (S51A, T57A, I122A, K161A and G174A) and showed surprisingly large effects. Differences in stabilization between the two sets of hydrogen bonds can be explained by the apparent gain of unwanted flexibility by reducing steric hindrance, for example affecting contacts in the cation-binding p-loop. Other mutations may lead to unwanted rigidity in the protein, leading to less stable contact formation in adjacent parts of the proteins (e.g., stacking interactions affected by G174A).

A loss of stacking interactions with the nucleotide ligands, proposed to be in the range of 16 kJ/mol (Gallivan and Dougherty, 1999; Huber et al., 2014), severely destabilizes APSK37. For the cation-π stacking interaction, the loss of binding energy translates directly into a similar loss in protein stability. Loss of π-π stacking resulted in marginally stable protein variants; however, the destabilization was less pronounced as expected for stacking interactions. The destabilization ranged from 3.1 kJ/mol for F91A to 7.2 kJ/mol for F175A. Targeting interactions of the p-loop residues K55 and T56 with the Mg^2+^ cation translated to the largest destabilization of the protein of up to 19.2 kJ/mol (K55A).

In summary, our data showed that substrate and co-factor binding determine the APSK37 in-cell protein stability. The spread in protein stability ΔG_f_^0^ from -10.7 to +8.5 kJ/mol is remarkable. Considering that the effect of the solvent water on the thermodynamic stability range is unknown, these values may be a lower limit of the actual effect on the protein. They may even be more precisely determined in a homogeneous *in vitro* environment using calorimetric measurement approaches such as isothermal titration calorimetry. Still, in the heterogeneous cellular environment the effects by far exceed the changes that had been reported previously for conditions such as osmotic pressure, cellular stress or differentiation (Stadmiller et al., 2017; Gnutt et al., 2019a): Hyperosmotic stress of *E. coli*, by adding 300 mM NaCl to regular buffer conditions, destabilized the expressed SH3 protein by ∼4.2 kJ/mol (Stadmiller et al., 2017). Cellular stress, induced by inhibition of the 26S proteasome using MG132, showed a destabilization of a SOD1-based folding reporter of ∼1 kJ/mol (Gnutt et al., 2019a). The SOD1-based folding reporter was also tested with regard to protein stability in differentiated and undifferentiated PC12 cells; it did not show significant changes in the standard folding free energies, but minor changes to thermal stability and folding properties (Gnutt et al., 2019a).

When comparing *in vitro* and in-cell results for different biomolecules, cellular crowding genuinely affects in-cell stability in a range of up to 7 kJ/mol as observed for cell surface antigen VlsE (Guzman et al., 2014) or SOD1 (Danielsson et al., 2015; Gnutt et al., 2019b), which are destabilized, an RNA hairpin (Gao et al., 2016) marginally affected and PGK (Dhar et al., 2011) which is stabilized in cells. Quinary interactions, that lead to (intracellular) fifth order structure of proteins change the conformational stability by up to ∼4.7 kJ/mol (Cohen and Pielak, 2016). Compared to the aforementioned cases of in-cell protein stability and their modulation by up to 7 kJ/mol, our study shows that ligand binding is a crucial factor to determine the in-cell stability of a protein, as indicated by the pronounced stability effects of 19.2 kJ/mol. It adds a novel descriptor to the recently discussed effects of quinary interactions and crowding, largely exceeding these effects in the systems studied so far. For this specific protein an interaction with its binding partners becomes stability-wise just as crucial as the induced folding of intrinsically disordered proteins by ligand binding (Fuxreiter and Peter, 2012). Ligand binding induced stability shifts will also be crucial to consider for marginally stable proteins that participate in liquid-liquid phase separation (Samanta et al., 2021).

What are the implications of the stability changes of the enzyme for the catalytic cycle? The APS kinase domain of PAPS synthase undergoes a series of ligand binding and releasing events during its catalytic cycle (Sekulic et al., 2007a; Ravilious and Jez, 2012; Brylski et al., 2019) (Figure 4A). Our in-cell results at ATP-depleted conditions show that the APS kinase free of bound substrates is highly unstable - as we have assigned the unfolding event at ∼17.5°C to this form due to its pronounced instability. Proceeding in the cycle, the kinase then preferentially binds either ATP or ADP (Ravilious and Jez, 2012) leading to a re-structuring within the otherwise flexible lid region (P151-S180) (Harjes et al., 2005; Sekulic et al., 2007b). This conformational change opens the APS/PAPS binding site for binding (Sekulic et al., 2007b). The severe destabilization of the R158A as well as the T56A and K55A mutations (Figure 2B) show, that the occupation of the ATP/ADP binding site and the resulting conformational change are essential for an initial stabilization to a marginal stability level. The K55 and T56 side-chains are responsible for the Mg^2+^ cation coordination. The R158 side chain interacts via cation-π-stacking with the adenine base of ATP/ADP and has been attributed to the structuring of the lid region of APSK (Sekulic et al., 2007b). The pronounced stabilization induced by the second ligand-binding event of APS/PAPS is best explained by the additive effect of ADP and APS we have observed *in vitro* (Figure 1F). In summary, during the catalytic cycle the actual stability of the APSK is subjected to temporal changes ranging from intrinsically unstable in its apo-form as well as without any substrate bound to marginal and great stabilization upon substrate binding in its two nucleotide binding sites.

As ATP fuels the PAPS cycle, physiological ATP concentrations will keep the PAPS synthase fraction in stable binding conformations, while ATP depletion should shift these fractions to less stable ones (Figure 4). Further, the intrinsic instability of the protein devoid of substrates and single-bound ligand complexes also sheds new light on the inhibitory PAPS synthase complex with ADP and APS bound (Brylski et al., 2019). The complex might not have only evolved as an inhibitory complex regulating activity but in addition may serve as a stable storage form of the APS kinase during ATP depletion (Brylski et al., 2019). The proposed storage complex is also a less ATP-dependent conformation in which effectively ADP and APS show only three phosphorylation equivalents, compared to ATP-APS and ADP-PAPS complexes having one more phosphorylation equivalent. The second unfolding event that we observed at high temperatures during ATP depletion of cells indicates that the ADP-APS binding conformation may be maintained for a fraction of the protein in cells at times of low ATP levels.

Temporal stability changes and control over pathways by ligand concentration changes are factors to consider when it comes to the properties of marginally stable proteins, such as PAPS synthases, inside cells. As the APS kinase plays a crucial role in the generation of the unique activated sulfate PAPS and catalyzes the rate-limiting step in PAPS biosynthesis (Grum et al., 2010), the temporal stability changes during the catalytic cycle and changing cellular ATP concentration observed suggest that sulfation pathways may be regulated by intracellular ATP levels. Decreasing ATP concentration under starvation or stress may destabilize key ATP-dependent enzymes to slow down metabolic activity. Thereby, the abundance of ATP in cells may be utilized to maintain ATP levels beyond what is needed for energy supply and to use it to additionally control metabolic activity. The excess pool may be used to stabilize proteins such as PAPS synthases and provide a ‘regulatory buffer’ in which ATP changes may be tolerated by cells.

In summary, we show APS kinase (APSK) stability and therefore its activity is highly determined by several factors including ligand binding capability and intracellular ATP levels. We engineered the FRET-based folding sensor APSK37 and used it for alanine-scanning mutagenesis of the ligand binding site. This revealed that the potential protein-ligand complexes have different impact on the overall protein stability. Further, pharmacological in-cell depletion of ATP levels inside cells confirmed, that APSK stability is directly connected to the accessible ATP as all ligands binding to APSK are generated from it. This connection has major implications for the catalytic cycle of the APSK kinase enzyme, which experiences significant changes in its stability during catalysis. The second unfolding event observed, suggests that in times of ATP depletion, the inhibitory ADP-APS complex may still be populated as a storage form to protect a fraction of APSK from aggregation and degradation. Our results show that the stability of catalytically active proteins inside cells may be primarily determined by ligand and co-factor binding, in addition to crowding effects and quinary interactions that determine the in-cell stability of proteins. This suggests a novel regulatory layer of catalytic activity by co-factor and ligand dependent stability regulation of the involved enzymes. This idea may be particularly applicable to enzymes that show marginal stability in the cell and are highly stabilized by ligand binding. The proposed mechanism will initiate further studies on different kinases and their associated regulatory complexes.

## Data Availability

The original contributions presented in the study are included in the article/Supplementary Material, further inquiries can be directed to the corresponding authors.
