# Supplementary figures and images for: Cellular ATP Levels Determine the Stability of a Nucleotide Kinase
Source: Front Mol Biosci. 2021 Dec 13;8:790304. doi: 10.3389/fmolb.2021.790304 (PMC8710738; doi:10.3389/fmolb.2021.790304)

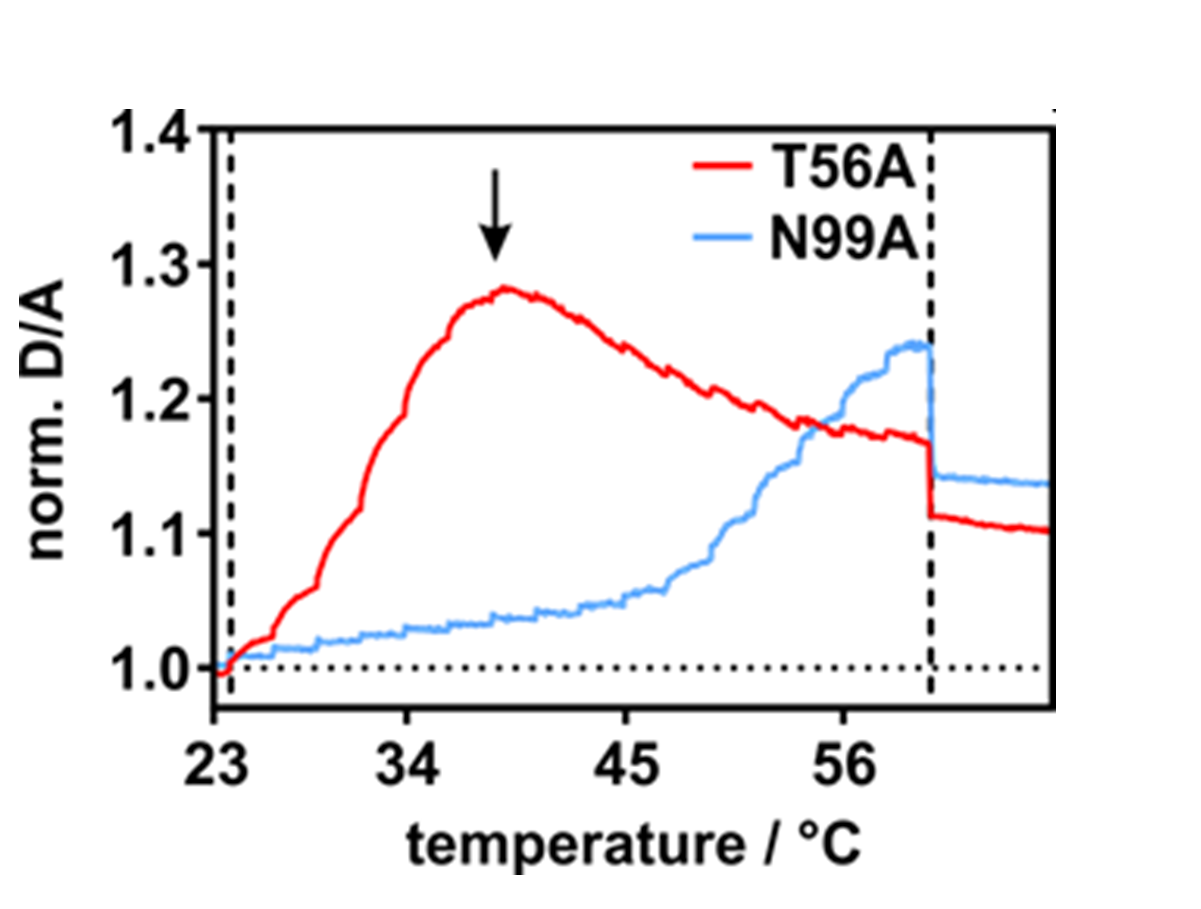

Supplement: Supplementary file 1 [file Image6.TIF]

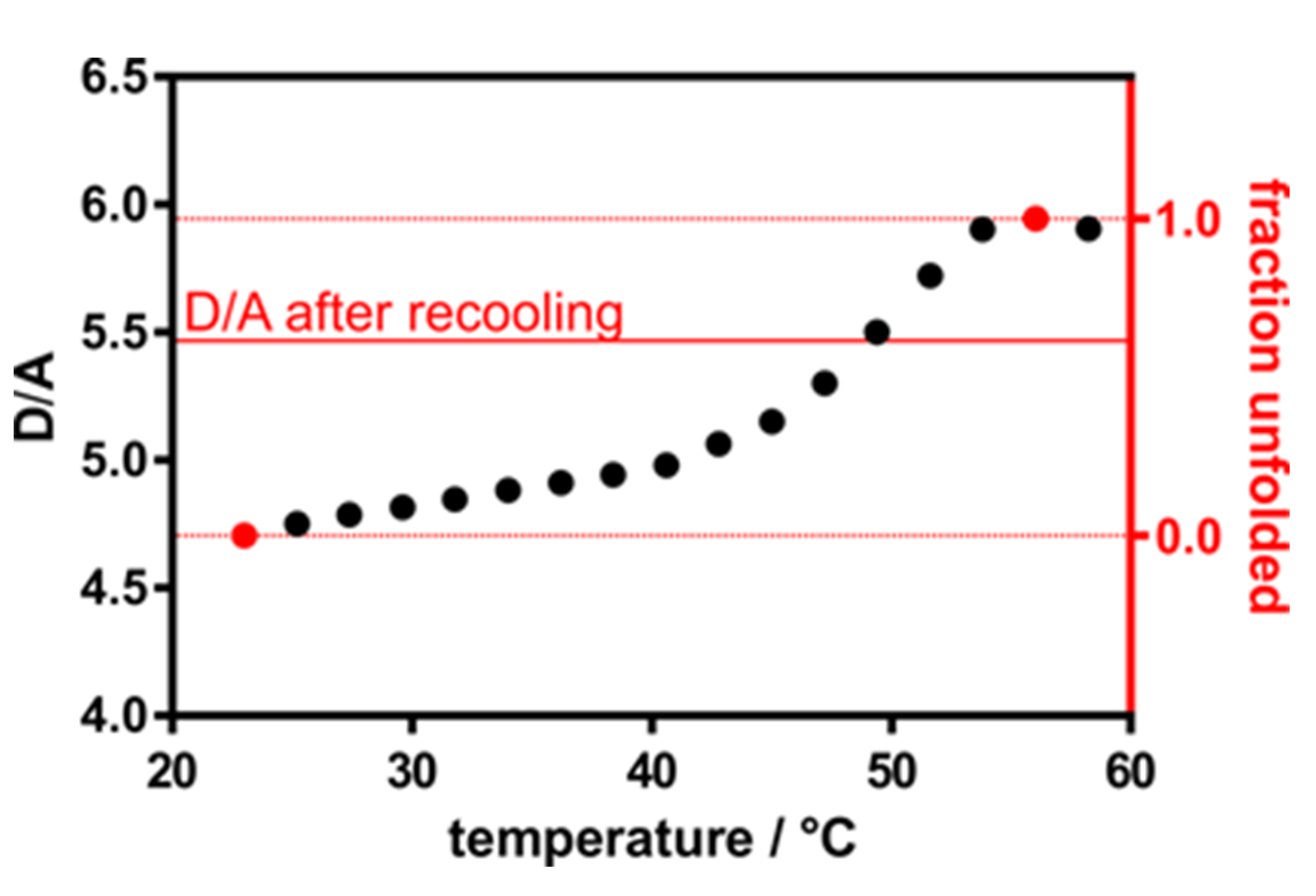

Supplement: Supplementary file 2 [file Image3.TIF]

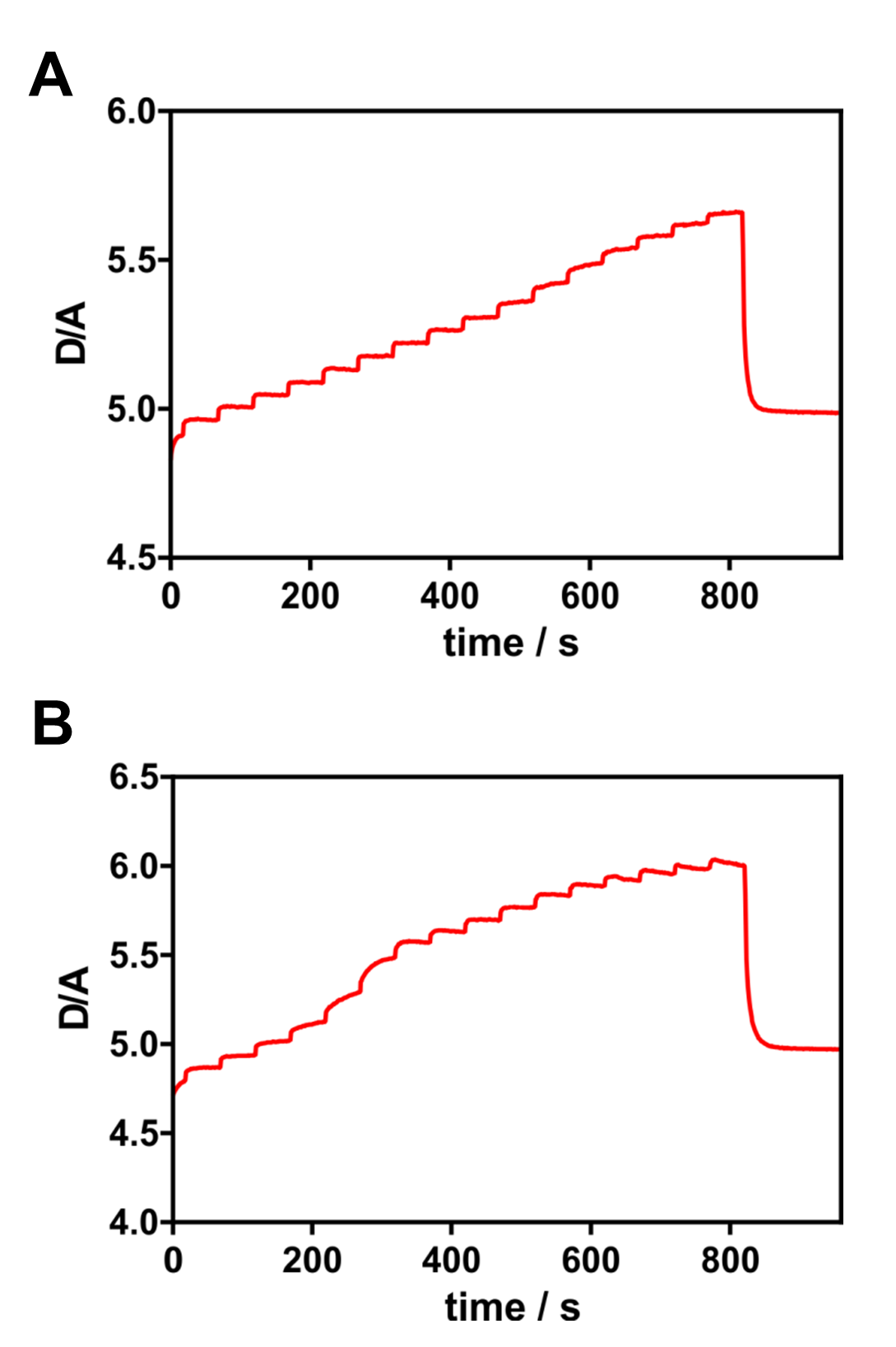

Supplement: Supplementary file 3 [file Image4.TIF]

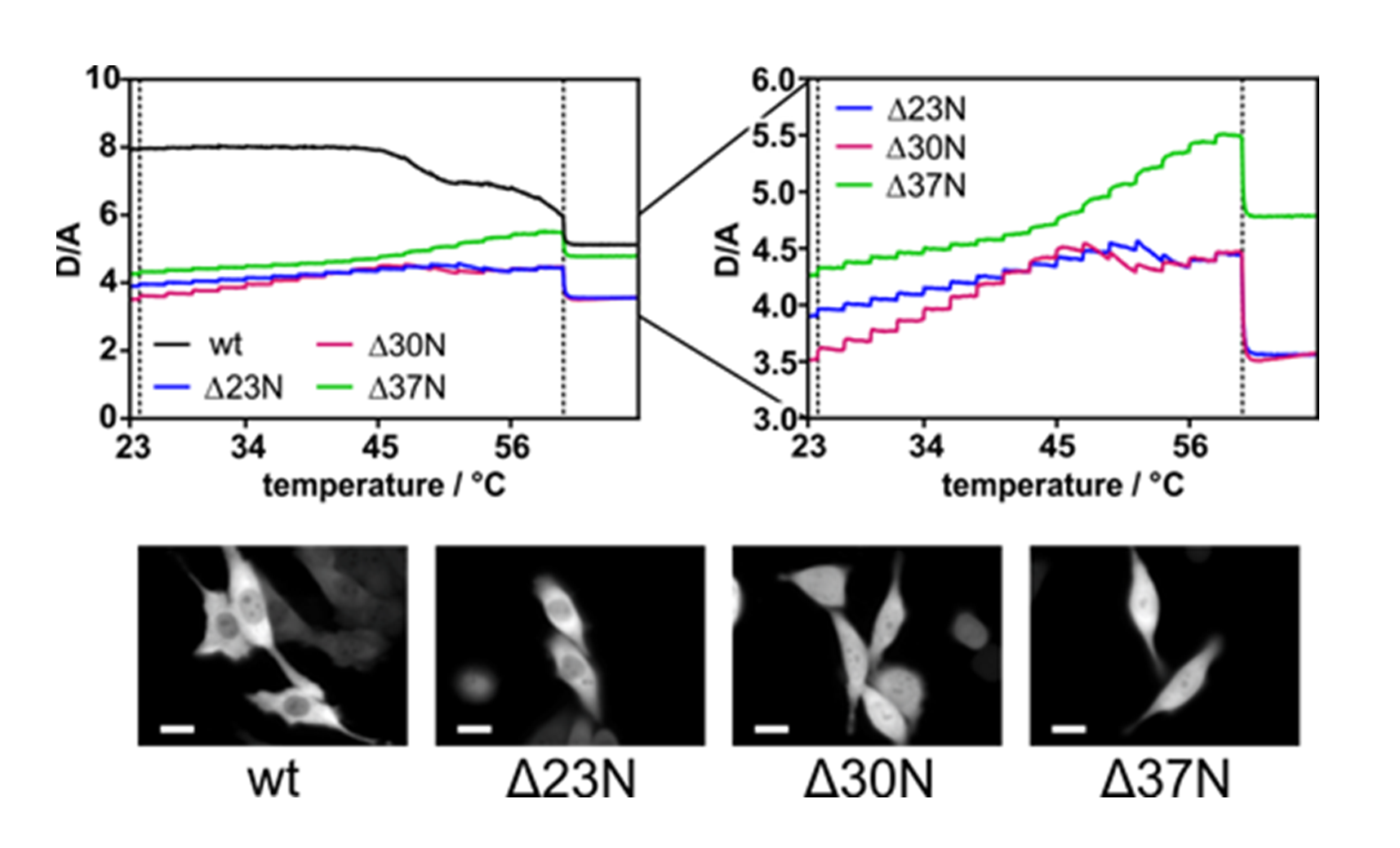

Supplement: Supplementary file 4 [file Image2.TIF]

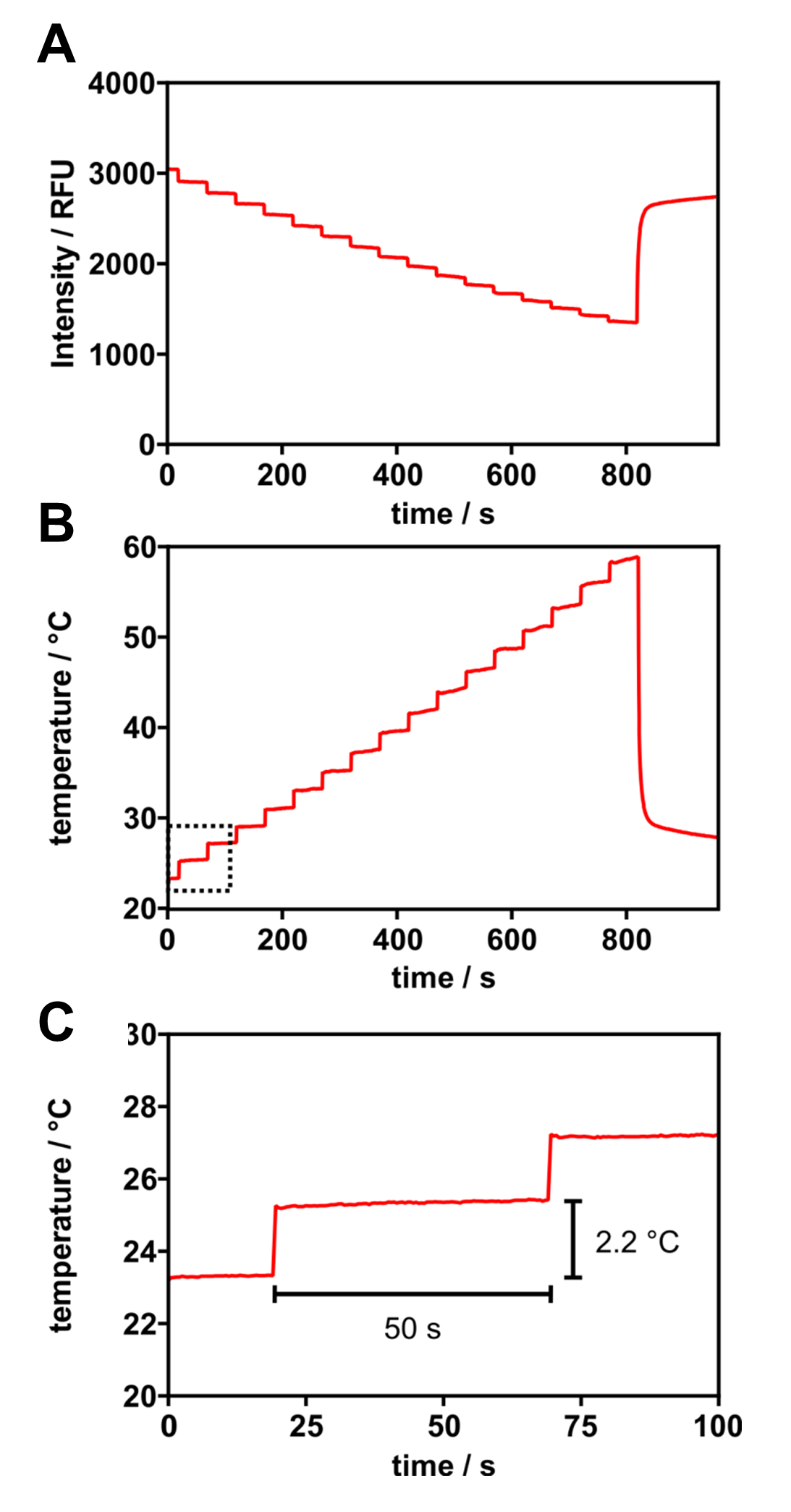

Supplement: Supplementary file 5 [file Image1.TIF]

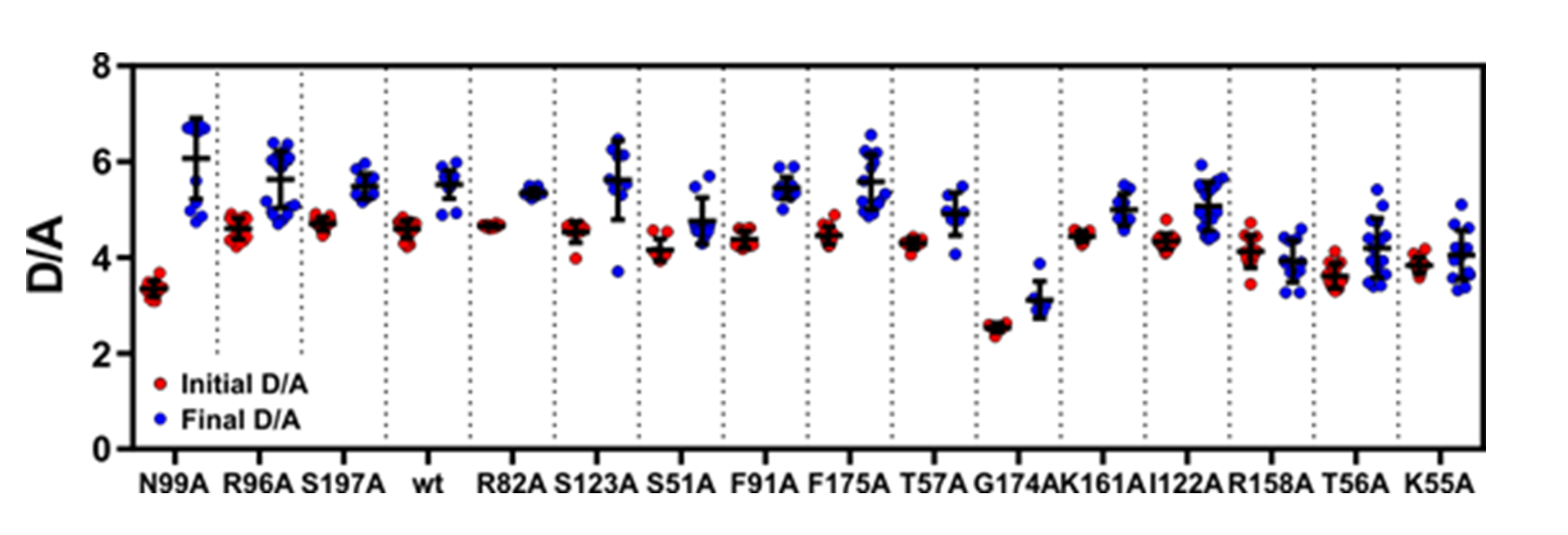

Supplement: Supplementary file 7 [file Image5.TIF]
