# Supplementary material for: Cellular ATP Levels Determine the Stability of a Nucleotide Kinase
Source: Front Mol Biosci. 2021 Dec 13;8:790304. doi: 10.3389/fmolb.2021.790304 (PMC8710738; doi:10.3389/fmolb.2021.790304)
Supplement: Supplementary file 6 [file DataSheet1.PDF]

**Figure S1: Temperature calibration using a Rhodamine B solution.** (**A**) Intensity time-trace during application of 16 mid-infrared temperature jumps and subsequent temperature relapse after laser turn-off. (**B**) Conversion of intensity data via calibration-equation published by *Büning et al* (Büning et al., 2017). Dashed square highlights the zoom-in. (**C**) Zoom-in of the first two temperature jumps.

**Figure S2: Comparison of N-terminal deletion of APS kinase domains.** (**Top**) Exemplary D/A traces of in-cell measurements of wild type and N-terminal deletions of APS kinase. Zoom-in of the deletions for better visualization. Dashed lines indicate start and end-point of temperature jumps. (**Bottom**) Exemplary fluorescence images of cells expressing wild type and N-terminal deletions of APS kinase. White bar for scale (20 µm).

**Figure S3: Unfolding recovery of APSK37.** Equilibrated D/A values of single temperature jumps are plotted against temperature. The minimum of the native baseline (first red data point) and the maximum of the unfolding baseline (second red data point) are defined as fully folded and fully unfolded. Solid red line indicates the equilibrated D/A after recooling. Unfolding recovery is determined as $[D/A_{unfolded}-D/A_{recooling}]/[D/A_{unfolded}-D/A_{folded}]$ and ~37 % in this specific example of APSK37 and ~30 % on average.

**Figure S4: D/A traces of the recombinantAPSK37.** (**A**) D/A time-trace of wild-type APSK37 (10 µM) during a temperature titration experiment. (**B**) D/A time-trace of wild-type APSK37 (10 µM) with 10 mM APS present during *Fast Relaxation Imaging* experiment.

**Figure S5: Initial and Final D/A of APSK37 mutants.** Data points depict single cells measured. Bars refer to mean +- s.d.

**Figure S6: Comparison of unstable and stable alanine mutants of APSK37.** Time x-axis was transferred into a temperature-scale for better visualization of data. Depicted temperature reflects the equilibration temperature of the respective T-jump. Arrow highlights the beginning of the negative baseline after unfolding of the protein.
